# Osteonecrosis of the jaws in 194 patients who have undergone intravenous bisphosphonate therapy in Spain

**DOI:** 10.4317/medoral.20092

**Published:** 2015-02-07

**Authors:** Carmen Vidal-Real, Mario Pérez-Sayáns, José-Manuel Suárez-Peñaranda, José-Manuel Gándara-Rey, Abel García-García

**Affiliations:** 1 DDS, Oral Medicine, Oral Surgery and Implantology Unit. Faculty of Medicine and Dentistry; 2 PhD, DDS. Oral Medicine, Oral Surgery and Implantology Unit. Faculty of Medicine and Dentistry.Instituto de Investigación Sanitaria de Santiago (IDIS), Santiago de Compostela, Spain; 3 MD, PhD. Department of Pathology and Forensic Sciences. University Hospital and School of Medicine of Santiago de Compostela; 4 MD, PhD, DDS. Oral Medicine, Oral Surgery and Implantology Unit. Faculty of Medicine and Dentistry; 5 MD, PhD. Oral Medicine, Oral Surgery and Implantology Unit. Faculty of Medicine and Dentistry. Instituto de Investigación Sanitaria de Santiago (IDIS), Santiago de Compostela

## Abstract

**Background:**

Osteonecrosis of the jaw (ONJ) is a destructive bone process in patients undergoing bisphosphonate therapy and it is modulated by local and systemic factors. The purpose of this article is to determine the prevalence of ONJ in patients who have undergone intravenous bisphosphonate therapy, and relate the risk factors described to establish a protocol to reduce the risk of developing ONJ.

**Material and Methods:**

We performed a retrospective study on 194 patients treated with IV bisphosponates, analyzing clinical and pathological variables.

**Results:**

The prevalence of ONJ was 12.9 %. The most remarkable complication was pain, which was reported by 80% of patients. The average age of the patients undergoing bisphosphonate therapy was 68.91 years. Most of non-diabetic patients did not develop ONJ (92.3%) (*p*=0.048). During bisphosphonate therapy, 3.1% of patients underwent extractions in the same percentage in the maxilla and in the mandible; all of which, except for one patient, developed ONJ (*p*<0.001). In regards to the periodontal state, 94.3% of patients without periodontal problems did not develop ONJ (*p*=0.001). Almost 50% of the necrosis were located unifocally on the mandible (*p*<0.001). The number of affected patients and the aggressiveness of the disease increased significantly three years after starting treatment (*p*<0.001).

**Conclusions:**

Etiology still is a controversial issue and we should focus on known risk factors, such as the development of surgical procedures in patients undergoing bisphosphonate therapy, especially in patients who have already started their treatment, a group in which ONJ prevalence increases. Moreover, a bad periodontal state in these patients is also an important risk factor, and the control of diabetes reduces it. Due to the above, all patients should be diagnosed and educated in oral hygiene prior to treatment, performing periodical maintenance, to detect possible traumatisms and periodontal infection as soon as possible.

**Key words:**Osteonecrosis of the jaws, intravenous bisphosphonate therapy.

## Introduction

The term osteonecrosis of the jaw (ONJ) was used for the first time in 2003 in a series of cases with 36 bone lesions of the mandible or maxilla in patients undergoing treatment with pamidronate or zolendronate. But it wasn’t until 2007 that a definition and standardization of the process was established by the American Association of Oral and Maxillofacial Surgeons (AAOMS). The Association describes it as a destructive bone process in patients undergoing bisphosphonate therapy who show bone exposure of over 8 weeks of evolution without former radiotherapy treatment (1,2).

The pathophysiology of bisphosphonate-related osteonecrosis is still under debate in international literature. Different theories have been described and an extensive list of mechanisms has been proposed. The two most studied hypotheses are the suppression of bone remodeling and angiogenesis (3). Although many studies and revisions mention the suppression of bone remodeling when referring to bisphosphonate-related osteonecrosis, there are currently changes in this hypothesis regarding three aspects. Firstly, osteonecrosis cases that are unrelated to the use of bisphosphonates have been described, in which other anti-remodeling agents have been used; secondly, bisphosphonate-related ONJ only occurs in the jaw and not in long bones or vertebrae; thirdly, IV administration of bisphosphonates increases the risk of osteonecrosis (4)

The risk factors that can contribute to the appearance of ONJ derive from the administration of bisphosphonates, systemic factors and local factors (5,6). The most relevant systemic factor is the presence of a malignant process, which is usually breast cancer, multiple myeloma or prostate cancer. Other influencing factors are diabetes, osteoporosis (in oncology patients) and medication related to steroids, immunosuppressive and antiangiogenic drugs (5).

Lastly, local factors are also related to the appearance of ONJ, such as oral hygiene and overall periodontal state. In most cases of bone necrosis, it appears after dental extractions, implant placement or lesions in the mucosa (7). The literature links dental extractions at the beginning of ONJ as one of the most common factors in bone necrosis in patients undergoing bisphosphonate therapy (approximately 86%) (8).

The purpose of this article is to determine the prevalence of ONJ in patients who have undergone intravenous bisphosphonate therapy, and relate the risk factors described to establish a protocol to reduce the risk of developing ONJ.

## Material and Methods

We performed a retrospective study on 194 patients, of which 139 (71.6%) were men and 55 (28.4%) were women, with an average age of 68.91, a standard deviation of 11.69 and a range between 42 and 93 years. All of the patients visited the Master of Oral Medicine, Oral Surgery and Implantology of the Faculty of Medicine and Dentistry of Santiago de Compostela. The patients were derived from the Oncology Unit of the ComplejoHospitalarioUniversitario of Santiago de Compostela (CHUS), for a dental examination prior to IV bisphosphonate therapy. All the patients who were submitted to IV bisphosphonates over a period of 7 years (2006-2013) were included in this study. This study has been approved by the Regional Ethics Committee Investigation of Galicia (2014/304) and all patients have received the consent informed.

All patients were treated with zoledronic acid. The main exclusion criteria was a history of treatment with radiotherapy. We performed a complete medical history, dental and mucosa examination. As part of the protocol, we collected data on the patients regarding treatment, dental status and evolution.

We analyzed the oral status of the patient at the time by performing a complete odontograph, assessing the periodontal state, the use of removable prosthesis and the presence of torus or dental and bone plaques through additional tests, mainly orthopantomography.

The variables we collected included: hypertension, diabetes, treatment with cortisone and chemotherapy, presence or not of osteonecrosis, tooth extractions or other procedures before, during or after starting bisphosphonate therapy, presence of infection or teeth with unfavorable prognosis, general periodontal state and the use of removable prosthesis.The patients with established osteonecrosis, we classified by degree of affectation, following the guidelines of Bagán *et al*. (9)

Statistical analysis

The collected data was analyzed with the SPSS statistical system, version 20.0 for Windows. The discontinuous quantitative or discreet variables were analyzed through descriptive statistics, expressing the results in mean, deviation and standard. The frequency tables and percentages were used for qualitative variables.

For the study of the association of variables we employed the Chi Square test, the T-Student test or the Anova Factor test, depending on the application conditions. Values in which *p* ≤0.05 were considered statistically significant.

## Results

**Figure 1:**
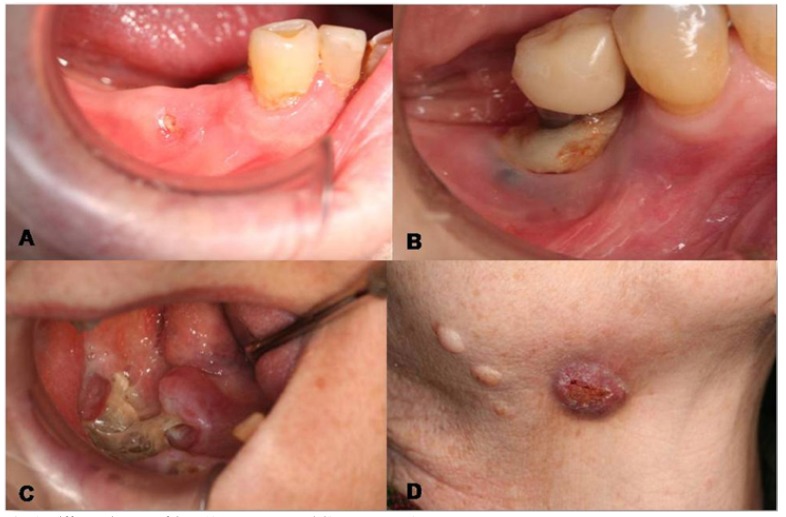
Different degrees of ONJ.A) Degree I. B and C) Degree II. D) Degree III.

Clinical and pathological characteristics of the sample and association analysis, are represented in  Table 1. Of the 25 patients (12.9%) who suffered ONJ, 8% had degree 0, 24% degree I, 72% degree II and 8% degree III. Hardly any differences were appreciated between genders, except for the fact that the 2 degree III patients were women. We can see on figure 1, some examples of the different degrees of ONJ.

**Table 1:**
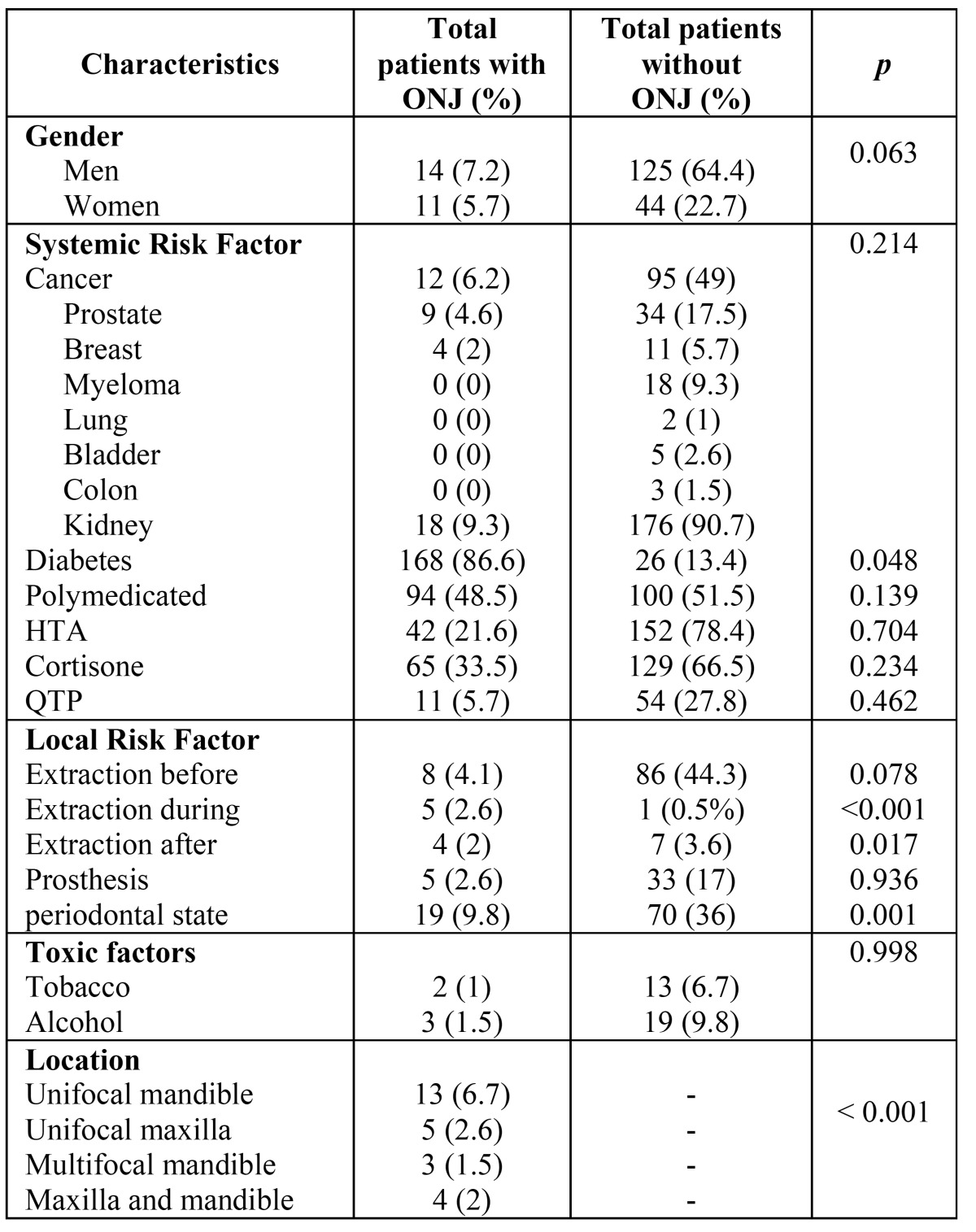
Clinical and pathological characteristics of the sample and association analysis.

The complications appearing in patients undergoing bisphosphonate therapy were only observed in the patients that developed ONJ; except for one patient who had a temporary paresthesia of the mental region of the 4th quadrant. The most remarkable complication was pain, which was reported by 80% of patients, followed by bone spicules (24%), abscesses (24%) and, in a lower degree, oroantral communication (4%) and extraoral fistula (4%).

The average age of the patients undergoing bisphosphonate therapy was 68.91 years (range 25 to 101 years), which is very similar to the age of ONJ patients (68.97 years), standard deviation 11.96, and a range between 42 and 93 years. There were no statistically significant differences (F =0.032, *p*=0.858). The gender of the patients did not show any statistical significant differences. Of the total study population of patients undergoing IV bisphosphonate therapy, 14 men (7.2%) and 11 women (5.7%) developed ONJ.

The reason for IV bisphosphonate therapy was prostate cancer in men (55.2%) and breast cancer in women (22.2%) followed by myeloma (9.3%) and lung cancer (7.7%), showing no differences between genders. All the patients were treated with zoledronic acid (Zometa®) at a 4mg dose and mostly in a monthly interval, except for one patient who received it quarterly.

In regards to systemic risk factors, most patients (86.6%) who developed ONJ were polymedicated, regardless of the consumption of drugs. The percentage of patients with hypertension (48.5%) as those treated with chemotherapy drugs (33.5%) and cortisone (21.6%) did not show any statistically significant differences. However, most of non-diabetic patients did not develop ONJ (92.3%) (X2=3.319, p=0.048).

In regards to dental extractions, 57.2% of the patients undergoing bisphosphonate therapy required dental extractions. 49% of the patients underwent tooth extractions before treatment, 13.4% of the upper jaw, 14.9% in the mandible and 20.6% in both jaws. During bisphosphonate therapy, 3.1% of patients underwent extractions in the same percentage in the maxilla and in the mandible; all of which, except for one patient, developed ONJ (X2=27.371, *p*<0.001). Once bisphosphonate therapy had ended, tooth extractions were performed in 5.7% of patients, the remaining 88.5% that did not undergo tooth extractions did not develop ONJ (X2=5.725, *p*=0.017). We must highlight that 17 of 25 patients that developed ONJ did not undergo tooth extractions, although the differences are not statistically significant.

As for local risk factors, of 38 patients with removable prosthesis, most of them (86.85%) did not suffer from ONJ and there were no statistically significant differences; while, in regards to the periodontal state (pocketsof >5mm and class II and III mobilities), 94.3% of patients without periodontal problems did not develop ONJ (X2= 10,488, p=0.001). Furthermore, most of the degree II ONJ patients (82.3%) and all of the degree III patients did have related periodontal problems.

As for related toxic factors, of the patients treated with bisphosphonates 7.7% were smokers, 11.3% had drinking habits and 75.8% combined both factors. 76% of ONJ patients were not smokers nor drinkers, although there were no statistically significant differences.

The most affected areas by osteonecrosis were the lower jaw, followed by the upper jaw, and in a lower percentage (16%) both jaws. Almost 50% of the necrosis were located unifocally on the mandible, compared with the unifocal necrosis of the maxilla, which only accounted for 20% (X2=187,319, *p*<0.001).

In regards to the period lapsing from the beginning of the bisphosphonate therapy to the appearance of the lesions, exposed bone areas started appearing after one year. The number of affected patients and the aggressiveness of the disease increased significantly three years after starting treatment. 24% of patients suffered from bone exposure during the first year of treatment, 32% at 2-3 years and 44% from 3 years onwards (X2=184,870, *p*<0.001).

## Discussion

IV bisphosphonates are commonly used drugs for the treatment of patients with bone metastasis to avoid pathological fractures and reduce pain. In regards to the type of bisphosphonate, it is important to distinguish the type, if it is nitrogenated or not, and the dose, duration and frequency of the treatment (cumulative doses) (5). The most relevant adverse effect described is osteonecrosis of the jaw, with a very variable prevalence according to the literature. It ranges between 0.7-12% (5,10,11), depending on the reviewed author, a similar figure as the one we obtained in our study (12.9%). Most ONJ processes are related to patients with prolonged treatments with nitrogenated IV bisphosphonates (5,6); furthermore, in regards to age and gender, we have observed a greater predisposition in advanced ages and women, since malignant diseases and osteoporosis appearing at the same time as breast cancer are more frequent in this type of patients (6).

All our patients received zoledronate (Zometa®), the bisphosphonate most frequently related to the appearance of ONJ, surely because it is the most powerful and extensively used third generation bisphosphonate (12-14). Bisphosphonates are mainly indicated for oncology patients; in our case the most prevalent cancer was prostate cancer in men (52.2%), breast cancer in women (30.4%) followed by multiple myeloma (17.4%), which showed no differences between genders. Our data differs from the reviewed literature (15-17) since the oncologist deriving most patients to our service is specialized in prostate cancer.

In regards to the doses, few studies include the exact doses of the patients, as well as the periods between doses. In our case, 91.3% of the patients received monthly doses of zoledronate, compared to 8.7% that received the doses quarterly. However, the literature concludes that higher doses of the drug increases the risk of developing ONJ (18,19). In regards to the period lapsing from the appearance of ONJ, once the bisphosphonate therapy has started, 24% of patients suffered from bone exposure during the first year of treatment, 32% at 2-3 years, and 44% from 3 years onwards. According to the study published by Bamias, the risk of suffering from ONJ in patients with IV bisphosphonate therapy increases from 39 months onwards, which coincides with the data collected in our study.

Other risk factors are also included, such as treatment with cortisone, obesity, smoking, diabetes or treatment with chemotherapy (13); in our study we did not find any statistically significant differences regarding these variables.

Tooth extractions were described as a precursory factor in 86% of the cases in which osteonecrosis appeared in patients treated with bisphosphonate therapy (10,20). Dental extractions before and after bisphosphonate therapy did not show statistical significance. However, 5 out of 6 patients who underwent extractions during the treatment with bisphosphonates developed ONJ.

We performed a conservative treatment with analgesics and antibiotics in the case of suppuration, in addition to a curettage and regularization of the areas with bone spicules. In those cases showing small areas of bone exposure (stage I) we proposed the use of a soft laser to vaporize the necrotic bone to reach the bleeding healthy bone. This minimally invasive technique allows us to create microperformations on the base of the bone and thus stimulate new vascularization. It also provides additional advantages such as its bactericide and biostimulation effect, while showing a better post-surgical recovery. We thus try to heal initial stages by trying to achieve a complete closure of the exposed bone (21,22).In the most advanced cases (degree II), we conducted a micro biota study to best establish the antibiotic treatment, although the period of treatment is not currently standardized (23,24). In the cases of degree III, surgical treatment was performed with bone resection and, in some cases, reconstruction of the area with a micro-vascularized flap. In all of the cases, we insisted on correct oral hygiene and the use of antiseptic mouthwash (clorhexidine at 0.12%). Our entire protocol is in agreement with the proposals of other authors of the reviewed literature (23,25).

Despite the treatment protocol proposed by several authors, most of them agree that the most important factor is prevention to avoid the appearance of osteonecrosis of the jaw (26). Several publications show that prevention reduces the risk of ONJ in patients undergoing IV bisphosphonate therapy (7,14,27). For this prevention, it is essential to conduct an extensive oral, dental and radio graphical assessment to detect bone or dental pathologies and treat them early on. Prevention by means of prior extraction of teeth with bad prognosis seems to be the appropriate measure. Of 94 patients in whom we developed this measure, 86 (91.5%) did not develop ONJ.

## Conclusions

Etiology still is a controversial issue in regards to the appearance of ONJ and we should focus on known risk factors, such as the development of surgical procedures in patients undergoing bisphosphonate therapy, especially in patients who have already started their treatment, a group in which ONJ prevalence increases. Moreover, a bad periodontal state in these patients is also an important risk factor in the development of ONJ, and the control of diabetes reduces it. Due to the above, all patients should be diagnosed and educated in oral hygiene prior to treatment, performing periodical maintenance, paying special attention to the lower jaw, to detect possible traumatisms and periodontal infection as soon as possible, which could eventually compromise healing and induce osteonecrosis later on.
